# New Therapeutic Targets in Autoimmune Cholangiopathies

**DOI:** 10.3389/fmed.2020.00117

**Published:** 2020-04-07

**Authors:** Alessio Gerussi, Martina Lucà, Laura Cristoferi, Vincenzo Ronca, Clara Mancuso, Chiara Milani, Daphne D'Amato, Sarah Elizabeth O'Donnell, Marco Carbone, Pietro Invernizzi

**Affiliations:** ^1^Division of Gastroenterology and Center for Autoimmune Liver Diseases, Department of Medicine and Surgery, University of Milano-Bicocca, Monza, Italy; ^2^European Reference Network on Hepatological Diseases (ERN RARE-LIVER), San Gerardo Hospital, Monza, Italy; ^3^National Institute of Health Research Liver Biomedical Research Centre Birmingham, Centre for Liver Research, Institute of Immunology and Immunotherapy, University of Birmingham, Birmingham, United Kingdom

**Keywords:** primary biliary cholangitis, primary sclerosing cholangitis, liver, FXR agonists, fibrates, microbiome, gut-liver axis

## Abstract

Primary biliary cholangitis (PBC) and primary sclerosing cholangitis (PSC) are autoimmune cholangiopathies characterized by limited treatment options. A more accurate understanding of the several pathways involved in these diseases has fostered the development of novel and promising targeted drugs. For PBC, the characterization of the role of farnesoid X receptor (FXR) and perixosome-proliferator activated receptor (PPAR) has paved the way to several clinical trials including different molecules with choleretic and antinflammatory action. Conversely, different pathogenetic models have been proposed in PSC such as the “leaky gut” hypothesis, a dysbiotic microbiota or a defect in mechanisms protecting against bile acid toxicity. Along these theories, new treatment approaches have been developed, ranging from drugs interfering with trafficking of lymphocytes from the gut to the liver, fecal microbiota transplantation or new biliary acids with possible immunomodulatory potential. Finally, for both diseases, antifibrotic agents are under investigation. In this review, we will illustrate current understanding of molecular mechanisms in PBC and PSC, focusing on actionable biological pathways for which novel treatments are being developed.

## Introduction

Autoimmune diseases of the biliary tract include primary biliary cholangitis (PBC) and primary sclerosing cholangitis (PSC). PBC and PSC are rare diseases of unknown etiology, immune-mediated pathogenesis and limited treatment options. Recently, there has been an increasing attention toward these rare diseases and novel agents are under investigation in clinical trials.

**Figure 1:**
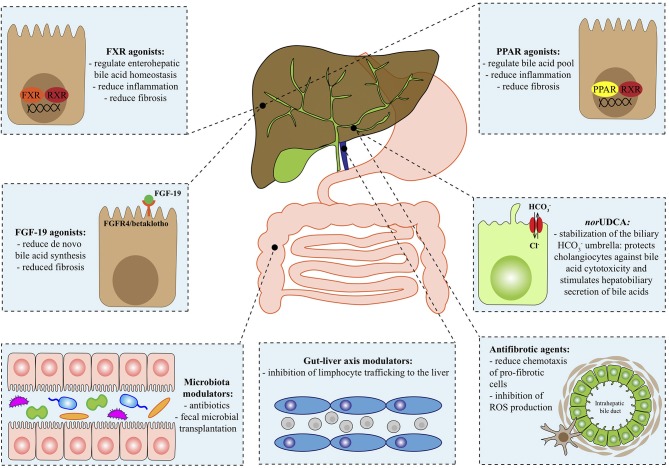
Therapeutical targets in autoimmune cholangiopathies. FGF-19, fibroblast growth factor 19; FGFR4, fibroblast growth factor receptor 4; FXR, farnesoid X receptor; HCO${}_{3}^{-}$, bicarbonate; norUDCA, norursodeoxycholic acid; PPAR, Peroxisome proliferator-activated receptor; ROS, reactive oxygen species; RXR, retinoid x receptor.

This review outlines the most promising novel agents for the treatment of PBC and PSC, adopting a target-driven approach: the biological target of each class of molecules is briefly summarized in the context of the pathogenesis of the disease and then preclinical and clinical results are presented (Figure 1 and Table 1).

**Table 1:** Novel pharmacological agents in autoimmune cholangiopathies.

	**Primary Biliary Cholangitis**	**Primary Sclerosing Cholangitis**
**FXR agonists**		
Bile acid		
- Obeticholic acid	✓	✓
Non bile acid		
- Tropifexor	✓	
- Cilofexor	✓	✓
- EDP-503	✓	
**FGF-19 agonists**		
- NGM-282	✓	✓
**PPAR agonists**		
- *PPAR-*α/(γ)- Bezafibrate/Fenofibrate	✓	✓
- PPAR-α/δ - Elafibranor	✓	✓
- PPAR-δ-Seladelpar	✓(*)	
**24-*****nor*****ursodeoxycholic** (*nor*-UDCA)		✓
**Antifibrotic agents**		
- Cenicriviroc		✓
- Setanaxib	✓	
**Immunological Agents**		
- Rituximab	✓	
- Ustekimumab	✓	
- Abatacept	✓	
- Baricitinib	✓(**)	
**Gut-Liver axis**		
- Vedolizumab		✓
- Antibiotics (Metronidazole, Vancomicin)		✓
- Timolumab		✓
- Fecal Microbial Transplantation		✓

(*) *Trial closed for adverse event*.

(**) *Trial closed in august 2019*.

*FXR, farnesoid X receptor; FGF-19, fibroblast growth factor 19; PPAR, Peroxisome proliferator-activated receptor; HCO${}_{3}^{-}$, bicarbonate; norUDCA, norursodeoxycholic acid*.

## Farnesoid X Receptor Agonists

Hepatocytes generate bile acids, i.e., cholic acid and chenodeoxycholic acid, from cholesterol through two pathways: most of the BAs are produced by the classical pathway, which involves the rate-limiting cholesterol 7alphahydroxylase (CYP7A1); the cytochrome (CYP) P450 27 alpha hydroxylase (CYP27A1) generates the remaining fractions. More recently, it has been reported that bile acids can also be generated by gut microbiota: these new bile acids are the phenylalanocholic acid, tyrosocholic acid and leucocholic acid (1).

Bile acids can exert different activities by binding to nuclear receptors, such as the farnesoid X receptor (FXR; NR1H4), pregnane X receptor, vitamin D receptor and Takeda G-protein-coupled receptor 5 (TGR5). FXR acts as transcription factor binding to FXR response elements in the DNA; it can operate as a monomer or together with retinoid X receptor (RXR; NR2B1) (2). FXR is mainly expressed in the liver and the gut, but can also be found in the kidney and adrenal gland, and its action exerts effects on the metabolism of bile acids, carbohydrates and lipids.

In the liver, FXR regulates bile acid synthesis, preventing their toxic accumulation. The bile salt export pump (BSEP) is expressed on the canalicular membranes of hepatocytes and promotes biliary excretion of bile acids. Its expression is dependent on FXR, while other transporters [e.g., multidrug resistance-associated protein (MRP) 3 and MRP4] are independent from FXR.

OSTα-OSTβ (*SLC51A* and *SLC51B*) is an heteromeric transporter expressed mainly in the distal portions of the gut and bile ducts, localized to the basolateral membrane of ileal enterocytes and biliary epithelial cells, respectively. Its main role is to transport bile acids across the membrane, but OSTα-OSTβ is also involved in steroids transport (3–5). FXR controls gene expression of OSTα-OSTβ, as proven by the marked reduction in OSTα and OSTβ expression in the ileum of Fxr-/- mice (6).

FXR induces the expression of the small heterodimer protein (SHP), also known as NR0B2 nuclear receptor subfamily 0, group B, member 2. SHP acts as transcription factor despite lacking a DNA binding domain, and inhibits CYP7A1 thanks to the recruitment of other proteins (mSin3A-Swi/Snf complex, G9a methyltransferase, the corepressor subunit GPS2) (7–10). CYP7A1 inhibition translates into a negative feedback inhibition of bile acid synthesis (2).

In pre-clinical setting, the activation of hepatic FXR is beneficial in reducing hepatic fibrosis, since it prevents toxicity due to accumulation of bile acids (11).

Among the FXR-agonists the main distinction is based on whether they are steroidal (i.e., bile acids) or non-steroidal agents. Limitations of steroidal agents are due to the intrinsic lipophilicity of FXR ligand binding site, which reduces solubility and bioavailability. Moreover, one of the most common side effects of steroidal FXR agonists is pruritus, which depends on the TGR5 agonistic properties (12). Nonetheless, also non-steroidal agents have shown mild pruritogen action despite not binding the TGR5 receptor. Interestingly, there is preliminary evidence that support the concept that non-steroidal FXR-agonists might have more anti-fibrotic effects (13).

**Obeticholic acid (OCA)** belongs to the class of steroidal FXR agonists and is a semi-synthetic bile acid. Currently, OCA is the only registered option for patients with PBC and incomplete response after 12 months of treatment with UDCA or intolerant to UDCA (14). The main side effect is itching, which is typically mild and seldom requires treatment withdrawal. Another side effect of OCA is the increase in low-density lipoprotein (LDL) cholesterol; the long-term significance of this side effect on cardiovascular risk is still unknown. OCA has been tested also in patients with PSC (AESOP trial), however final results are still waited.

### Non-bile Acid FXR Agonists

**Tropifexor** has been evaluated in a double-blind, randomized, placebo-controlled, phase 2 study in PBC, but only the interim analysis is available. Interestingly, to avoid confounding due to the possible FXR-mediated alkaline phosphatase (ALP) gene induction, gamma glutamyl transpeptidase (GGT) reduction was chosen instead of ALP reduction as primary endpoint of this trial.

The non-steroidal FXR agonist **Cilofexor** has been tested in PSC in a randomized, double-blind, placebo-controlled phase 2 trial, and although the primary endpoint was safety, it can be noticed that it significantly reduced ALP levels, especially in the 100 mg arm (15). A randomized, double-blind, placebo-controlled phase 2 trial in PBC and a phase 3 trial in PSC are currently undergoing.

**EDP-305** is another non-steroidal FXR agonist being evaluated in a randomized, double-blind, placebo-controlled phase 2 trial in PBC. In murine models of fibrosis EDP-305 reduced the extent of fibrotic areas assessed by morphometric quantification (16).

## Fibroblast Growth Factor 19 Agonists

During cholestasis, high levels of bile acids favor fibroblast growth factor 19 (FGF-19) expression. The increased concentration of FGF-19 in the gut stimulates activation of the FGFR4/betaklotho receptor in the liver. FGF19 then migrates to the liver where reduces CYP7A1 gene expression (7, 17). FGFR4 and betaklotho forms a cell surface receptor complex and represent the effectors of the liver activity of FGF19. FGF19 is also induced by FXR activation in the enterocytes.

Preclinical data have shown a higher risk of hepatocellular carcinoma (HCC) in transgenic mice with ectopic expression of FGF19 in the skeletal muscle of transgenic mice (18). Furthermore, 15% of human HCC cancers show co-amplification of FGF19 and cyclin D1 on 11q13.3 (19).

A synthetic analog **NGM282** was developed without carcinogenic potential and its safety and efficacy is under investigation in clinical trials. NGM282 has been tested in two randomized, double-blind, placebo-controlled trials in PBC and PSC, showing conflicting results. The PBC study lasted for 28 days while the design of the PSC study scheduled treatment for 12 weeks.

In PBC, NGM282 achieved a reduction of >15% of ALP levels in 50% of patients treated compared to 7% of the placebo group. Similar proportions were found between the 0.3 mg and the 3 mg arms. Main side effect was non severe diarrhea (20).

Conversely, in the PSC study of NGM282, ALP levels did not significantly drop in the 1 and 3 mg arms compared to placebo (21). We do acknowledge that the clinical significance of ALP in PBC and PSC is different, considering that PBC has a fairly stable and slowly progressing disease course where ALP is a good prognostic marker, compared to the erratic disease course of PSC, where ALP levels can intermittently rise and fall due to episodic cholangitis (22).

Interestingly, in both studies, NGM282 decreased the α-Hydroxy-4-cholesten-3-one (C4) levels in treatment groups confirming that this drug acts by directly inhibiting the *de-novo* bile acid synthesis through the classical pathway.

## Peroxisome Proliferator- Activated Receptors Agonists

Peroxisome proliferator-activated receptors (PPARs) are nuclear receptors of a family containing three isotypes: PPARα (NR1C1), PPARβ/δ (NR1C2) and PPARγ (NR1C3). After engagement with their ligand, PPARs form a heterodimer with the retinoid X receptor, binding to specific DNA sequences in the regulatory regions of target genes.

PPARα is highly expressed in tissues with marked fatty acid oxidation activity, including liver, heart and skeletal muscle, brown adipose tissue and kidney. Beyond its ability to regulate fatty acid catabolism in different conditions of food intake and starvation, animal models of atherosclerosis and non-alcoholic steatohepatitis have shown that PPARα has also anti-inflammatory properties. Moreover, PPARα agonism determines inhibition of bile acids synthesis by acting on CYP7A1 and cytochrome sterol 27-hydroxylase (CYP27A1). There is also evidence that PPAR-α abrogates the uptake of bile acids in hepatocytes through the inhibition of the basolateral transporter sodium-taurocholate-cotransporting polypeptide and up-regulates expression of human *MDR3* gene favoring canalicular export of phospholipids (23).

PPARβ/δ is expressed in hepatocytes, cholangiocytes, and non-parenchymal cells (Kuppfer cells, hepatic stellate cells). PPARδ is utilized by biliary epithelial cells to control bile components transporters (24) and favors the timely removal of apoptotic cells by Kuppfer cells, in order to prevent potential autoimmune phenomena to arise (25). PPAR-γ expression is mostly restricted to Kupffer cells (25). In cultured human biliary epithelial cells from patients with PBC there is a down-regulation of PPAR-γ and the activation of PPAR-γ is associated with reduced inflammation (26). A PPAR-γ agonist reduced portal inflammation in murine models of PBC (27).

**Figure 2:**
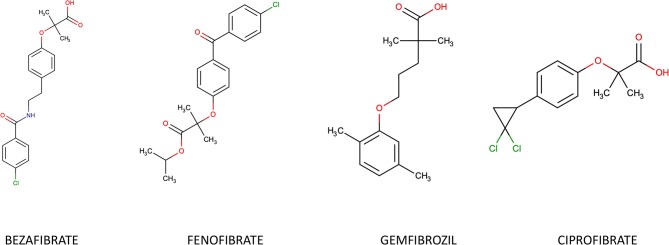
Chemical structure of most common fibrates.

Natural ligands of PPAR-α are derivatives of fatty acids generated during lipolysis, lipogenesis or fatty acids catabolism. Synthetic PPAR-α agonists belong to the group of fibrates, derivatives of fibric acid: Gemfibrozil, Fenofibrate, Ciprofibrate and Bezafibrate (Figure 2). They are typically used in treatment of isolated hypertriglyceridemia or mixed dyslipidemia. Fenofibrate is 10-fold more specific for the α- isoform compared to the γ- one, while Bezafibrate is considered a pan-PPAR-agonist due to its similar affinity for the three isoforms. Most of their actions are derived from their PPARα agonism.

There is growing evidence of the therapeutic efficacy of fibrates in PBC, while evidence is still limited for PSC. **Bezafibrate** has been evaluated in a 24 months, double-blind, randomized, placebo-controlled, phase 3 trial (BEZURSO trial), at the dosage of 400 mg per day in patients with incomplete biochemical response after 12 months of UDCA. The rate of ALP normalization was 67% in the treatment arm, compared to the 2% in placebo group. In addition, Bezafibrate did not worsen pruritus, which was even improved in a subset of cases, which is in line with available literature (28). The results from BEZURSO trial endorse the concept that fibrates reduce the production of bile acids in hepatocytes since patients in the 400 mg arm experienced a 70% drop in C4 serum levels, whereas patients in the placebo arm did not show any significant change. Conversely, experience on **Fenofibrate** in PBC derive from smaller cohorts (29–33). Both Bezafibrate and Fenofibrate show side effects typical of their pharmacological class, namely myalgias, transaminitis and increase in creatine kinase and creatinine. The long-term safety of these agents during treatment for cholestatic diseases is still to be ascertained. Since safety and efficacy profile of Bezafibrate and Fenofibrate seems to be similar, but Bezafibrate is supported by a randomized controlled trial, it would be reasonable to choose Bezafibrate as the fibrate of choice in PBC until new data are available.

There are now new molecules in the pipeline which show more selective PPAR-δ activity. Seladelpar is a selective PPAR-δ agonist (34), while Elafibranor is PPARα/δ.

Based on the safety information derived from two phase 2 trials (35), the potential efficacy of **Seladelpar** has been evaluated in the ENHANCE trial, a 52-week, double-blind, placebo-controlled, randomized, Phase 3 study. However, in November 2019, the ENHANCE trial was put on hold following the unexpected histological findings observed in the Phase 2b study of Seladelpar in subjects with non-alcoholic steatohepatitis (NASH). Atypical histological findings, including interface hepatitis and biliary injury were found in the planned interval biopsies.

**Elafibranor** has been recently evaluated in a multicenter randomized double-blind placebo-controlled phase 2 Study in PBC.

Literature supporting the use of fibrates in PSC is scanty. At the time of writing, no randomized controlled trials have been published, and retrospective case series come mostly from Japan (36, 37).

A French-Spanish retrospective study showed a 40% reduction in the levels of ALP after 12 weeks of treatment with fibrates [either Fenofibrate (200 mg/day) or Bezafibrate (400 mg/day)]. There were no major safety issues and authors cautiously support further studies. To us, it is conceivable to further investigate the potential benefit of fibrates in PSC, based on their inherent anticholestatic and anti-inflammatory properties.

## 24-*nor*ursodeoxycholic and the Biliary HCO${}_{3}^{-}$ Umbrella

The HCO${}_{3}^{-}$ umbrella hypothesis asserts that bicarbonate (HCO${}_{3}^{-}$) ions, secreted by cholangiocytes and hepatocytes, form a defensive barrier on the apical side of the hepatocytes (38). When this system is malfunctioning, glycine-conjugated bile acids become able to cross the cholangiocyte membrane, bypassing membrane transporters. This phenomenon leads to cholangiocyte apoptosis and senescence (39). There is evidence supporting the concept that in cholangiopathies like PBC and PSC the biliary HCO${}_{3}^{-}$ umbrella is defective (40, 41).

The biliary HCO${}_{3}^{-}$ umbrella can be stabilized through **24-norursodeoxycholic (nor-UDCA)**, mainly thank to its ability of undergoing cholehepatic shunting. *Nor*-UDCA is relatively resistant to *N*-acyl-amidation with taurine or glycine, so that cholangiocytes can reabsorb it and it can be re-secreted into bile after getting through periductular capillary plexus and hepatocytes. The importance of this process is the ability to cause a profound stimulus to bicarbonate secretion from cholangiocytes, strengthening the impaired HCO${}_{3}^{-}$ umbrella of cholangiopathies.

Mdr2-/- mice are typically considered a surrogate *in vivo* model of PSC (42). The lack of *Mdr2*-encoded membrane protein, which is a canalicular transporter deputed to transport phospholipids from hepatocytes to the bile, causes a cholangiocyte injury, due to bile acid toxicity from increased concentration of free non-micellar bile acids. In this model *nor*-UDCA increases the hydrophilicity of bile acids, stimulates bile flow and induces biliary transporters and detoxification enzymes (43).

A double-blind, randomized, placebo-controlled, phase 2 study evaluating *nor*-UDCA in patients with PSC has shown promising results in terms of safety of the molecule and reduction of serum alkaline phosphatase after 12 weeks of treatment (44). This trial assessed three different treatment regimens (*nor*-UDCA 500, 1,000 mg/day, and 1,500 mg/day) compared to a four arm with placebo. All doses showed a significant reduction in ALP levels, in a dose-dependent manner, and similar results were achieved for transaminases and gamma-glutamyl transferase levels. Pruritus did not occur more frequently in the treatment arms, and *nor*-UDCA showed a good safety profile.

A phase 3 clinical study is currently ongoing.

## Immunological Targets

PBC and PSC are both included in the group of autoimmune diseases of the biliary tract; however, while PBC is much closer to a typical autoimmune condition, PSC is a complex disease with aspects also typical of fibrotic and preneoplastic conditions. Aside from the classificatory debate, the immune system takes part of many pathogenetic processes of both PBC and PSC. Nevertheless, many strategies targeting immune cells have failed so far (45–53). Biological agents, like **Rituximab** (54, 55), **Ustekinumab** (56), and **Abatacept** (57) were studied in PBC, based on preclinical promising data (rituximab and abatacept) or stimulated by results from genome-wide association studies (ustekinumab), but conflicting results have been produced and these molecules are not part of the therapeutic armamentarium.

Among the novel agents under investigation, there is **Baricitinib**, a small molecule which is a reversible inhibitor of Janus kinase (JAK) 1 and 2. In mammals, different receptors are bound by members of the JAK family (JAK 1-3). The receptor-ligand binding determines a cascade of activation and modifications leading to the generation of docking sites for the SH2 domain of the cytoplasmic transcription factors termed signal transducers and activators of transcription (STATs). Several downstream signals are regulated by JAK and STAT proteins, comprising interleukins (ILs), interferons (IFNs), and the switch toward T helper (Th) 1, 2, or 17 of naïve T cells is finely regulated by a JAK-mediated signaling (58). Baricitinib is already included in the available registered drugs for the treatment of rheumatoid arthritis (59), and was supposed to be evaluated in a randomized double-blind, placebo-controlled, phase 2, study in patients with PBC and partial response to UDCA. Unexpectedly, in August 2019 Lilly, the company that developed the molecule, decided to terminate the trial.

## Antifibrotic Agents

Inflammation, cholestasis and fibrosis are tightly connected to determine the vicious cycle toward cirrhosis (60). Cholangiopathies show a biliary-type of fibrosis which usually spreads from the portal tract to the lobule (61). None of the registered therapies for PBC are inherently antifibrotic, but halt progression of the fibrotic process through antagonism of inflammation and/or cholestasis. However, specific antifibrotic therapies are urgently needed to potentially achieve fibrosis reversal, considered that a not negligible proportion of patients do progress despite effective treatment (62).

A novel agent located at the crossroad between inflammation and fibrosis is **Cenicriviroc**. Cenicriviroc targets C-C motif chemokine receptor (CCR) types 2 and 5, which are typically expressed by monocytes, Kupffer cells and hepatic stellate cells. There is mounting evidence that, upon liver injury, CCR2 and CCR5 together with their ligands favor macrophage recruitment infiltration and stellate cells activation (63, 64). In Bile-duct ligated and Mdr2*-/-* mice Cenicriviroc, together with another agent (all-trans retinoic acid), reduced bile acids, plasma liver enzymes and histological markers of necrosis and fibrosis (65). A randomized, double-blind, multinational, phase 2b enrolling patients with Non-alcoholic fatty liver steatohepatitis (NASH) revealed the antifibrotic capacity of Cenicriviroc, which halted fibrosis progression more frequently than placebo, without improving NASH-related liver inflammation (66). Cenicriviroc is currently under evaluation in PSC.

**Setanaxib** is another promising antifibrotic agent characterized by a completely novel molecular target and potentially representing a first-in-class molecule for the treatment of PBC and PSC. The main targets of Setanaxib are NADPH oxidase (NOX) proteins (67), which are enzymes over-induced in conditions of chronic stress, like chronic inflammatory and fibrosing diseases (68, 69). Activation of stellate cells to myofibroblasts is promoted by NOX1, NOX2, and NOX4 isoforms (70, 71).

In bile-duct ligation and NASH murine models, Setanaxib reduced histological markers of fibrosis through NOX-inhibition. Setanaxib has been recently evaluated in a multicenter, randomized, double-blind, placebo-controlled, phase 2 study in patients with PBC and incomplete response to UDCA. The primary endpoint was reduction in the levels of GGT, which is considered a more accurate marker of oxidative stress in the liver (72).

## Modulation of the Microbiota and Gut-Liver Axis

While PBC is classically limited to the small bile ducts of the liver, PSC also involves large ducts and is often associated with inflammatory bowel disease (IBD) (53). The involvement of the distal part of the biliary tree and the frequent co-existence of IBD have suggested that PSC may derive from the disruption of some physiological process in the gut. The focus has been put onto two players: the microbiota and the gut-liver axis.

There is a rising interest in the study of microbiota changes in pathological conditions (73–76). Dysbiosis, i.e., the abnormal composition of gut microbiota, has been described in patients across several diseases, included cirrhosis (77), PBC (78), and PSC (79). Dysbiosis in the gut can also involve viral and fungal species, as recently proved in IBD (80) and PSC (81). Germ-free Mdr2-/- mice show increased hepatitis and cholestatic injury compared to conventionally-housed Mdr2-/- mice (82). Nevertheless, it is still under debate whether dysbiosis has a causal relationship with these pathological findings in humans.

From a therapeutic point of view, modulation of microbiota in PSC represents a fascinating option, since some data suggest that the progression of the disease might be due to a vicious cycle of inflammation and fibrosis driven by translocation of pathobionts from the gut to the liver (83). It is likely that tackling this process could be of benefit, and there is some clinical evidence to support this concept. The use of antibiotics (e.g., **Metronidazole**, **Vancomycin**) in patients with PSC can reduce blood markers of liver injury and cholestasis, despite data on long-term benefit are lacking (84–86).

Recently, the provoking concept that non-communicable diseases (i.e., those not caused by infectious microbes) might be communicable via the transfer of microbiota has been proposed (87). This challenging theory is supported by several line of experimental data showing that if a dysbiotic microbiota is transferred from diseased animals to healthy mice the latter will develop the disease (88). Conversely, there is also evidence that **Fecal Microbial Transplantation (FMT)** may be of benefit for many diseases (89, 90), in addition to the established indication for recurrent Clostridium Difficile infection (91). Indeed, a seminal open-label study has shown the capacity of FMT to augment microbiota diversity in patients with PSC. In a subset of individuals FMT improved biochemical markers of cholestasis; however the small number of individuals included in this pilot study (i.e., 10 patients) obviously prevents solid conclusions on efficacy (92).

Regarding gut-liver axis, the anatomical link between gut and liver translates also in an intimate interconnection at the molecular level, with several substances flowing to the liver through the portal vein system. When intestinal permeability is higher than normal, bacterial byproducts reaching the liver are potential drivers of inflammation and fibrogenesis. Two main barriers control this process: the epithelial barrier and the gut-vascular barrier (93). Under physiological conditions, mucosal addressin cell adhesion molecule (MAdCAM)-1 is expressed on gut endothelium and is not expressed in the liver, avoiding recruitment of T cells in the liver (94). In PSC there is evidence of aberrant expression of MAdCAM-1 in the liver (95). Despite theoretically promising, the use of **Vedolizumab**, which antagonize MAdCAM-1 and its receptor α4β7 and it is approved for IBD treatment, did not show efficacy in patients with PSC and IBD (a phase 3 trial was retired in 2018). A retrospective study on Vedolizumab in patients with PSC and IBD also failed to reveal a positive signal (96).

The increased expression of MAdCAM-1 in the liver is mainly due to the activation of vascular adhesion protein (VAP)-1, which is an enzyme involved in amine oxidation. Intrahepatic and circulating levels of VAP-1 are increased in patients with PSC compared to non-PSC individuals, and a single-arm, open-label, multi-center trial evaluating the efficacy of an anti-VAP-1 molecule, **Timolumab**, is currently ongoing (97).

## Conclusions

Many novel drugs are currently under investigation for PBC and PSC. The potential future availability of many novel agents opens the challenge for the identification of the right candidate for each specific drug or combination of drugs based on the mechanism of action and safety profile.

It is key the improvement of risk stratification strategies for this purpose (98), which will require deep, longitudinal phenotyping of individuals by means of multi-omics analysis including the exposome, along with the microbiome, genome, metabolome, among the others (99–104). Then, we need to implement algorithms to proficiently integrate these big data to cluster patients across different phenotypes and trajectories of the disease (105, 106); for this, the collaboration with data science professionals and experts in artificial intelligence will be fundamental (107, 108). The last part of this process will be to put in practice clinical trials with different, multimodal treatment strategies (109).

## Author Contributions

This work was done in full by AG. ML has contributed to a part of the contents. CMi and DD'A have created and processed the image. LC has created the table. All authors contributed to manuscript revision, read, and approved the submitted version.

### Conflict of Interest

The authors declare that the research was conducted in the absence of any commercial or financial relationships that could be construed as a potential conflict of interest.
